# Supplementary material for: High Incidence of Strawberry Polerovirus 1 in the Czech Republic and Its Vectors, Genetic Variability and Recombination
Source: Viruses. 2021 Dec 11;13(12):2487. doi: 10.3390/v13122487 (PMC8706236; doi:10.3390/v13122487)
Supplement: Supplementary file 1 [file viruses-13-02487-s001.zip › viruses-1495965-supplementary_proofread.pdf]

High incidence of strawberry polerovirus 1 in the Czech Republic and its vectors, genetic variability, and recombination – Fránová et. al (**SUPPLEMENTARY MATERIAL**)

Figures:

Figure S1. Localities of sampling in the Czech Republic.

Figure S2. *F. vesca* 'Alpine' (No. 814) infected with strawberry polerovirus 1 (SPV1), strawberry mottle virus (SMoV) and strawberry crinkle virus (SCV) with mosaic, severe leaf malformation and dwarf symptoms.

Figure S3. *Fragaria vesca* 'Alpine' plant infected with SMoV showing severe mosaic on the 35th day post inoculation (dpi) with *Aphis gossypii*.

Figure S4. Irregular vein clearing and leaf curl of F. vesca 'Alpine' plant infected with SPV1 and SCV at 32 days post inoculation with *Chaetosiphon fragaefolii* (source of inoculum: *F. vesca* cv. Rujana 7/2017).

Figure S5. Phylogeny of aa sequences for particular genes of SPV1.

Figure S6. Phylogeny of nt and aa partial sequences of the P1-P2 gene (1600 nt fragment) of SPV1.

Figure S7. Analysis of potential recombination events in P1 and P5 genes between available SPV1 isolates.

Tables:

Table S1. Overview of the tested plants: symptoms, virus detection and Sanger sequencing of SPV1 isolates originating from strawberry plants from different localities of the Czech Republic.

Table S2. Description of all primers used in the study.

Table S3. Results of RT-PCR determination of SPV1 in *Aphis gossypii* batches and recipient *Fragaria vesca* 'Alpine' plants after transmission trials.

Table S4. Synonymous vs. nonsynonymous nucleotide mutations in putative recombinant regions of the P1 (A) and P5 (B) genes.

Figure S1. Localities of sampling in the Czech Republic. Numbers and region codes correspond to **Table S1**

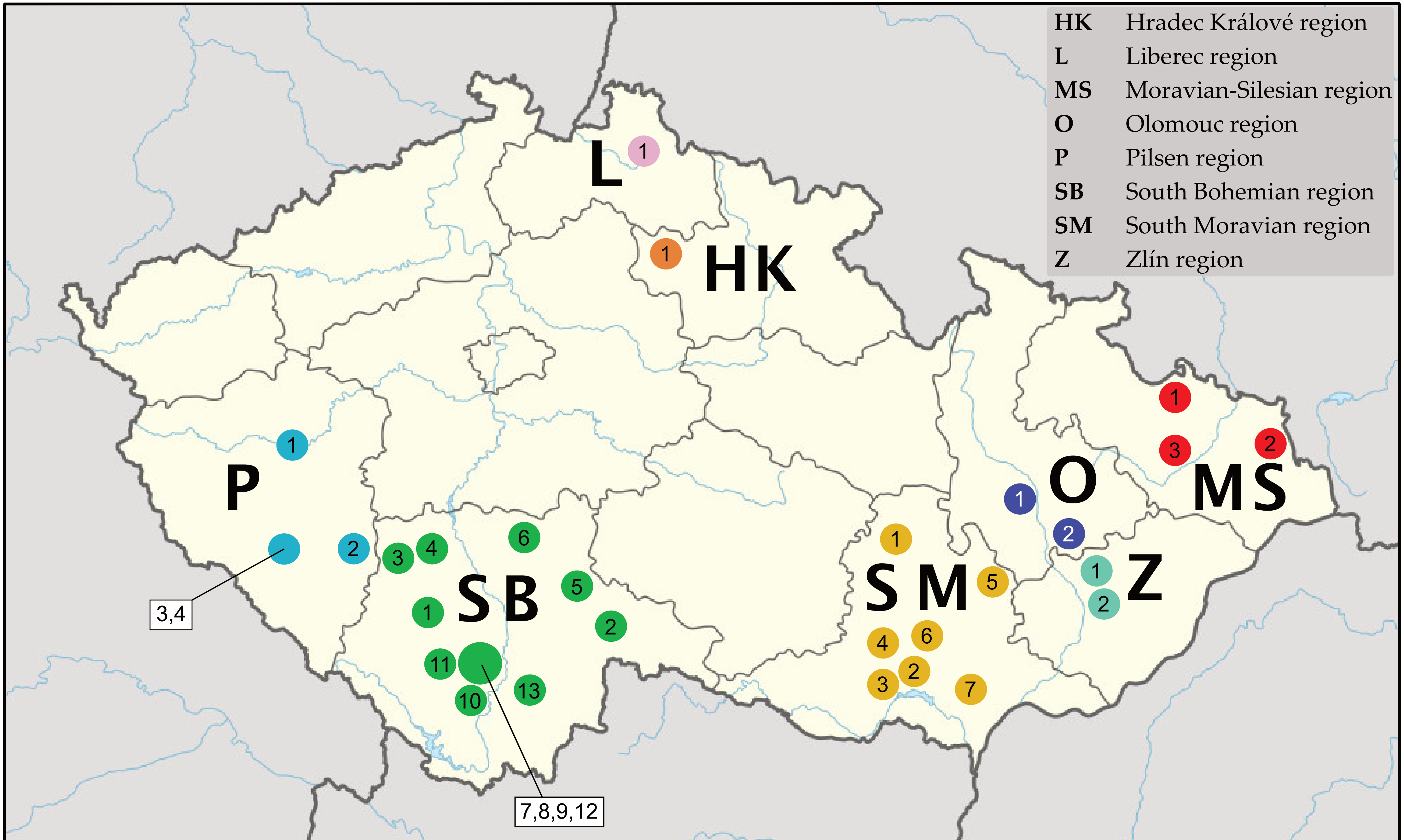

Map was created using original work licenced under Creative Commons licence (NordNordWest, Creative Commons CC BY-SA 3.0 de, hÆps://commons.wikimedia.org/w/index.php?curid=42830552).

Figure S2. *F. vesca* 'Alpine' (No. 814) infected with strawberry polerovirus 1 (SPV1), strawberry mottle virus (SMoV) and strawberry crinkle virus (SCV) with mosaic, severe leaf malformation and dwarf symptoms.

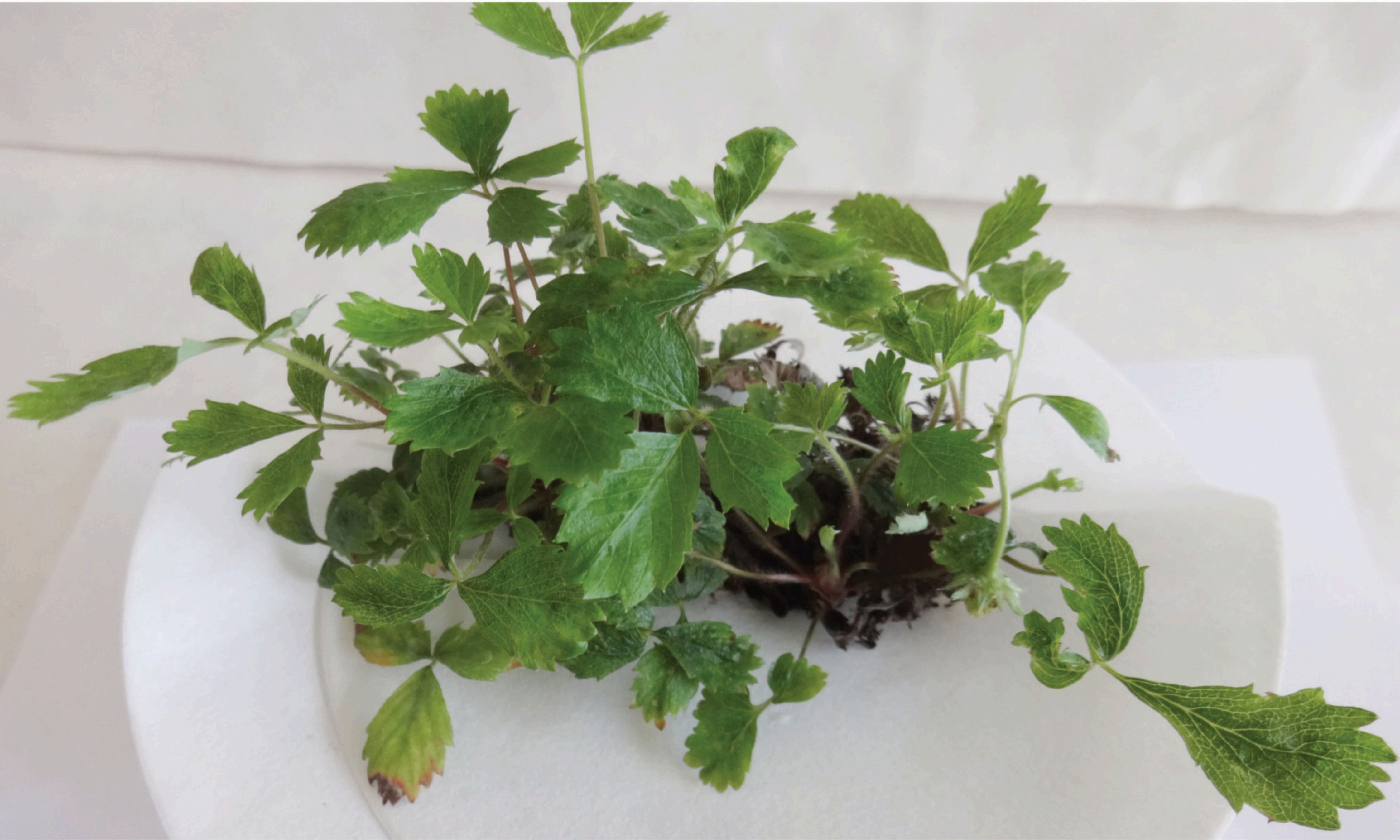

Figure S3. *Fragaria vesca* 'Alpine' plant infected with SMoV showing severe mosaic on 35 dpi with *Aphis gossypii.*

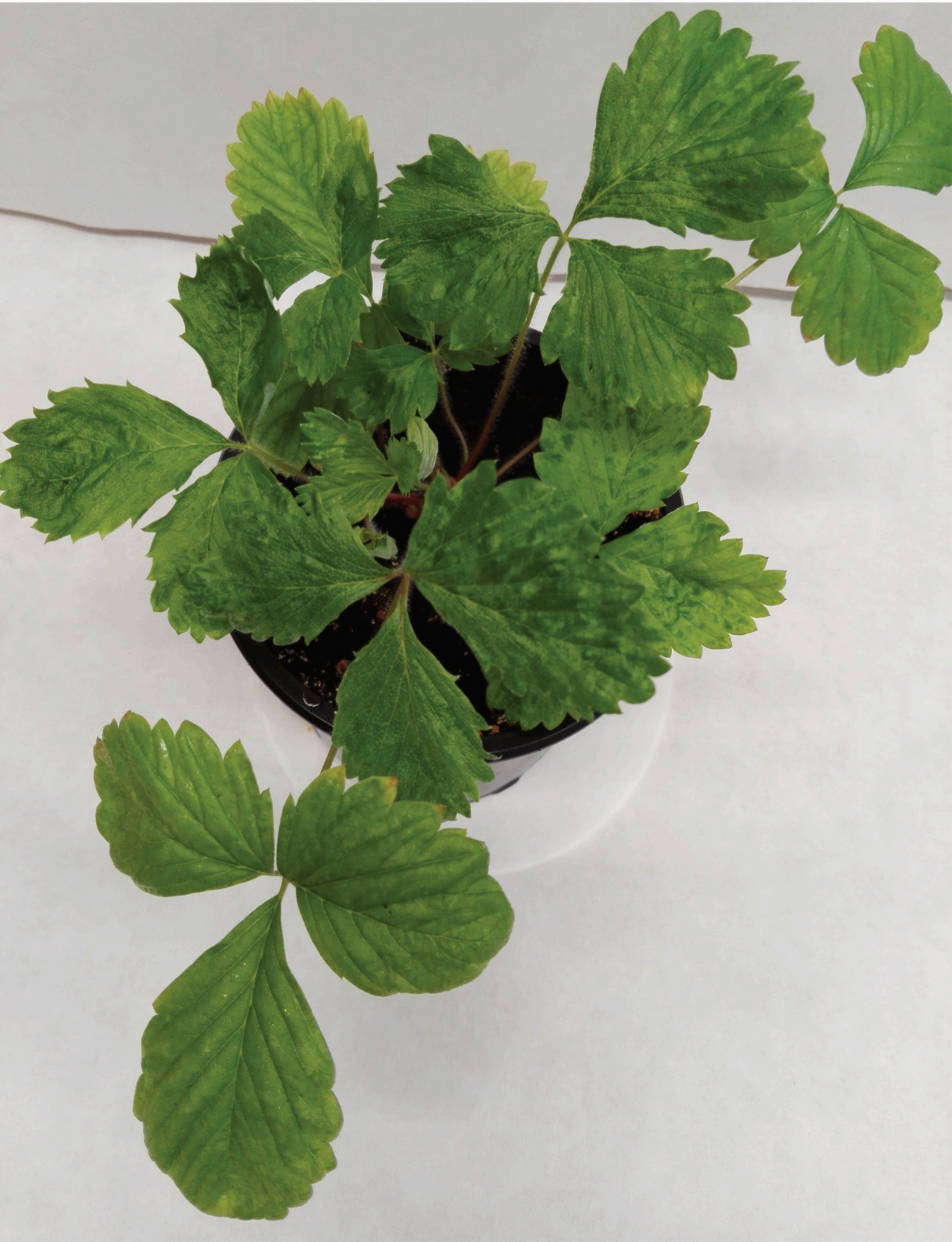

Figure S4. Irregular vein clearing and leaf curl of *F. vesca* 'Alpine' plant infected with SPV1 and SCV at 32 dpi with *Chaetosiphon fragaefolii*
(source of inoculum: *F. vesca*)

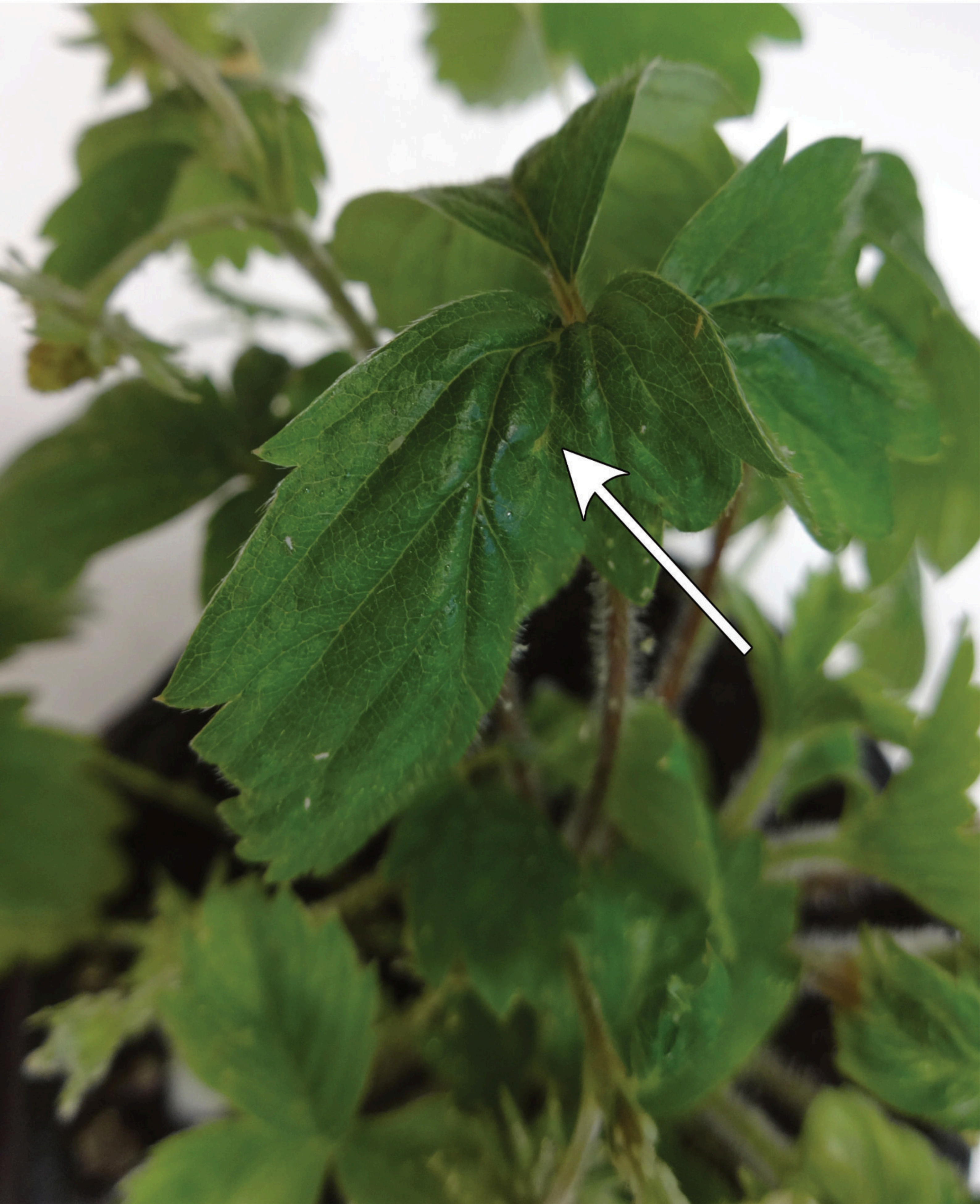

Figure S5. Phylogeny of aa sequences for particular genes of SPV1. Only GenBank accessions with complete genome/CDS were used. Isolates sequenced in this study are in color.

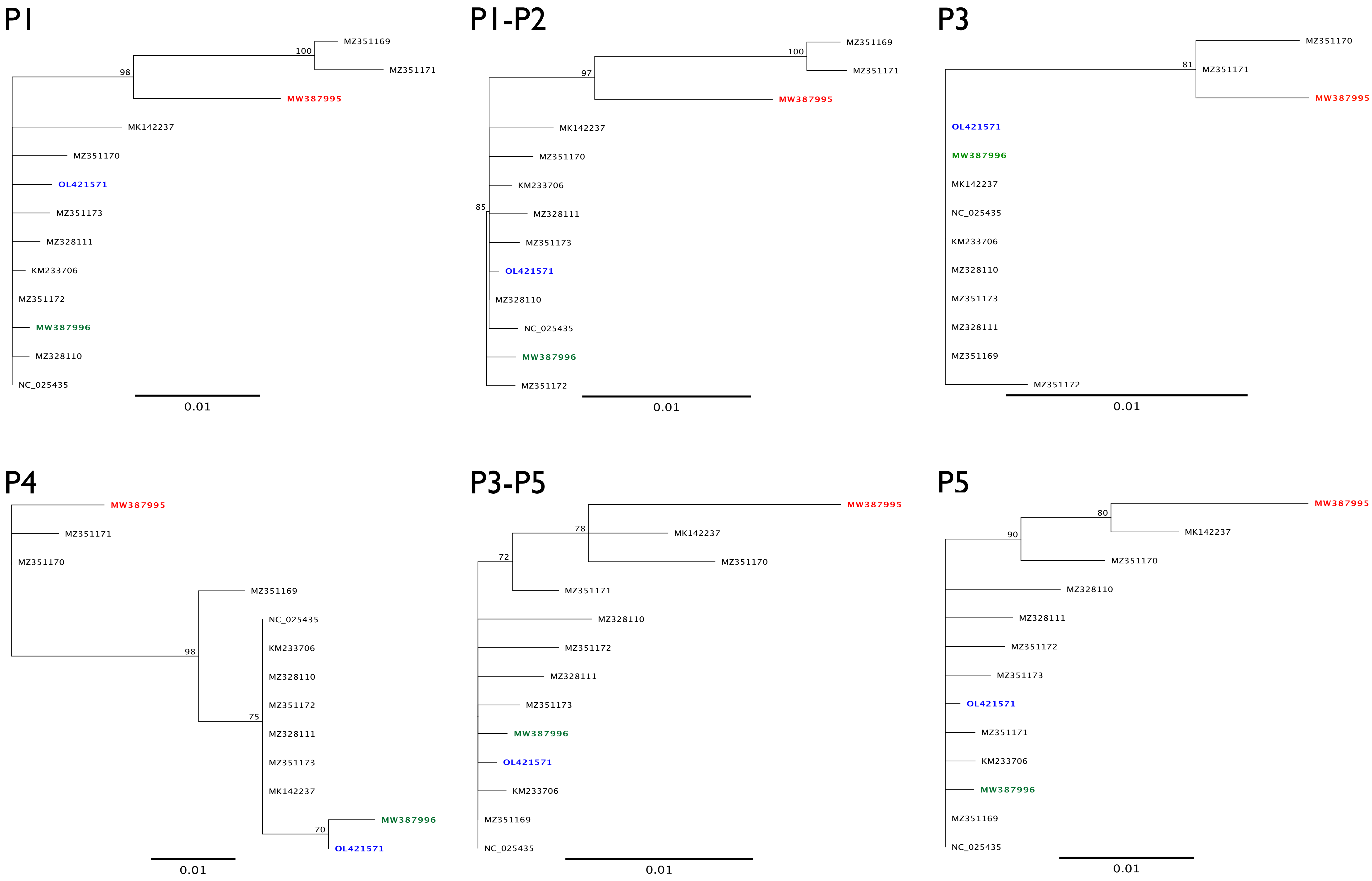

Figure S6. Phylogeny of nt and aa partial sequences of P1-P2 gene (1600 nt fragment) of SPV1. Isolates sequenced in this study are in bold; color depicts isolates for which complete CDSs were obtained (colors are in the same color as in Fig. S5).

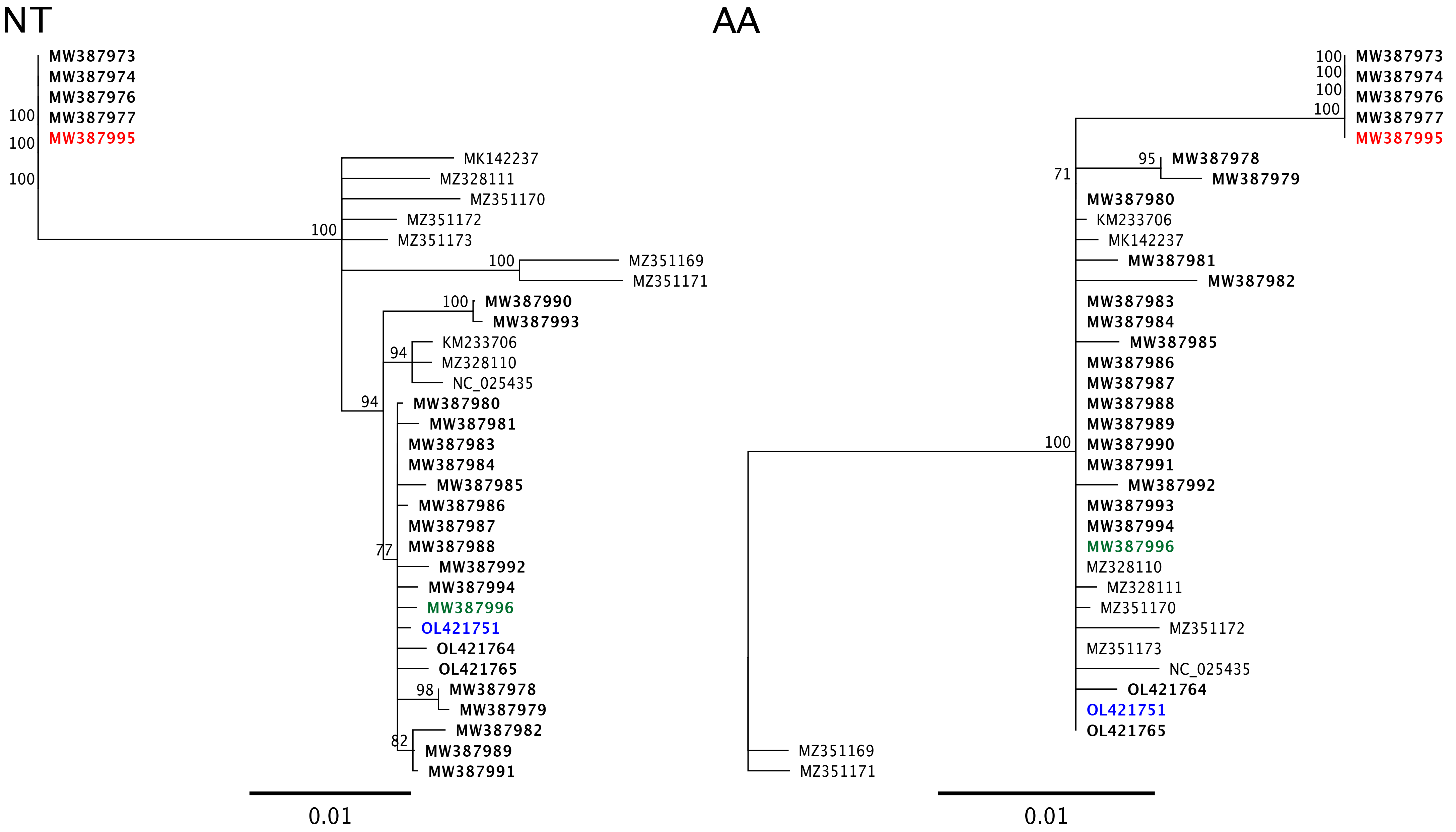

Figure S7. Analysis of potential recombination events in P1 and P5 genes between available SPV1 isolates: (A) overview with nucleotide changes depicted; (B) RDP5 software recombination graphs and p-values obtained; (C) phylogenetic trees of recombined and non-recombined regions of particular genes. Nucleotide changes were evaluated against reference sequence NC_025435 and are depicted as black (any nucleotide) or color (red = A, blue = C, green = T, yellow = G) vertical lines on each sequence.

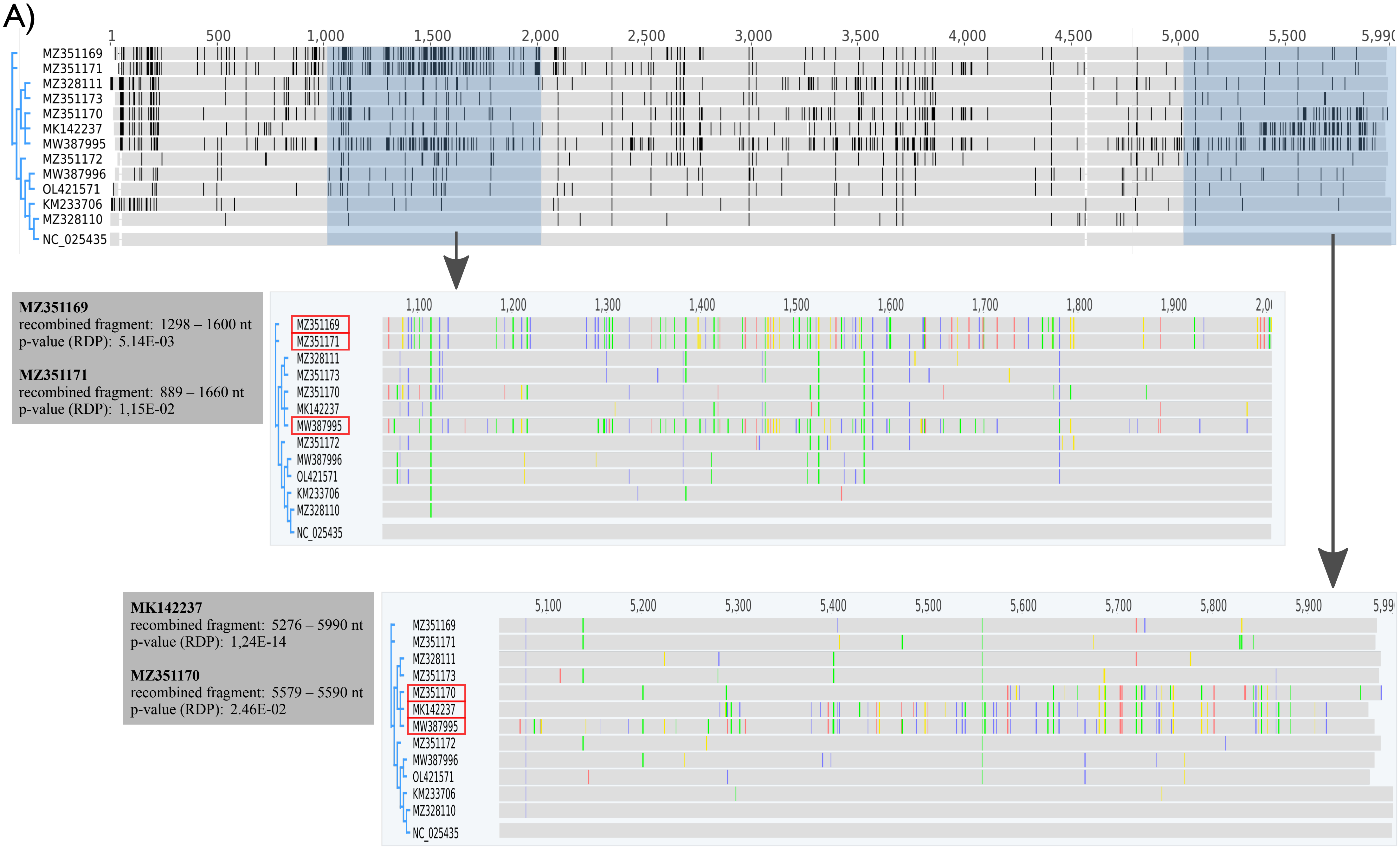

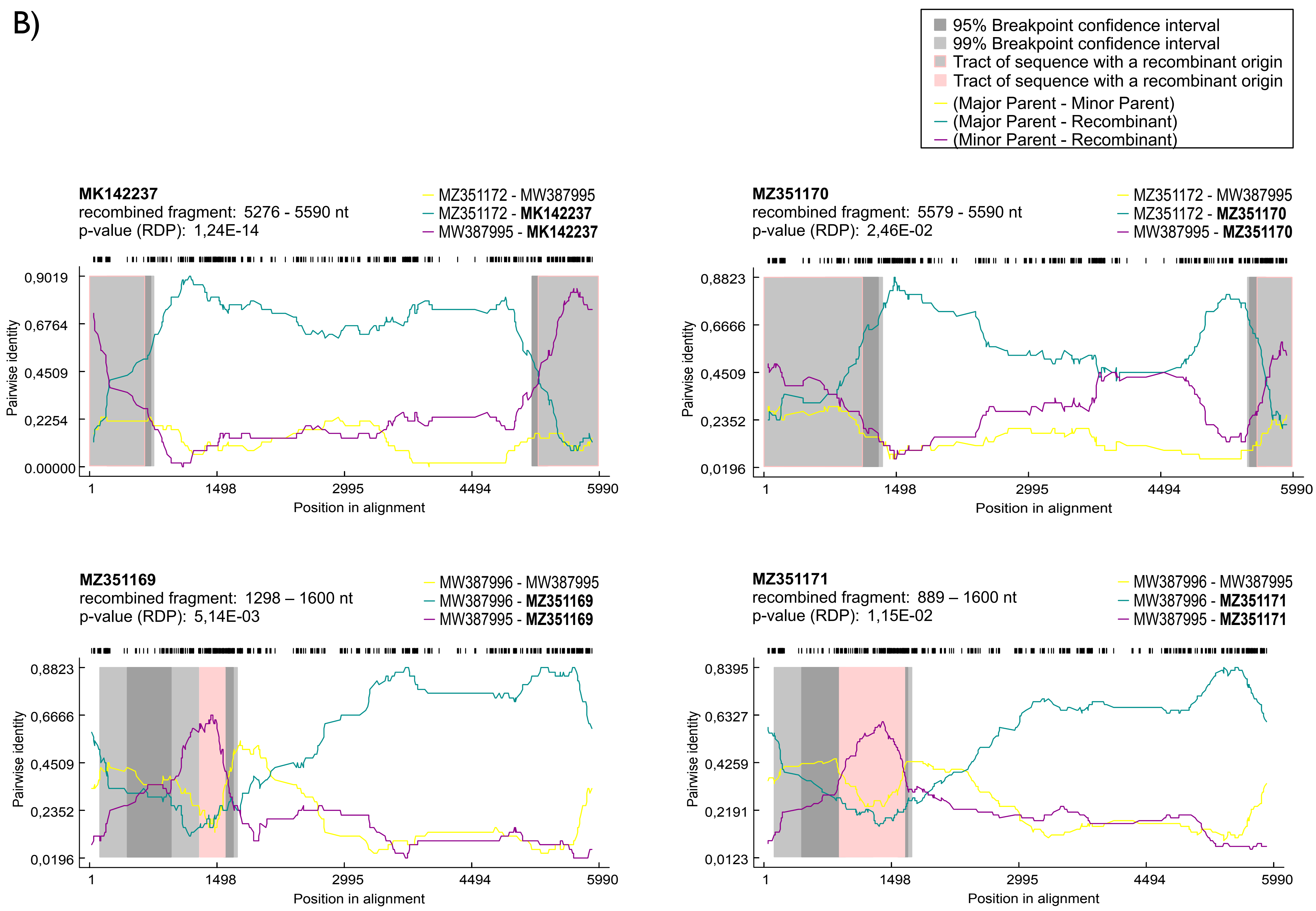

B)
95% Breakpoint confidence interval
99% Breakpoint confidence interval
Tract of sequence with a recombinant origin
Tract of sequence with a recombinant origin
(Major Parent - Minor Parent)
(Major Parent - Recombinant)
(Minor Parent - Recombinant)
MK142237
recombined fragment: 5276 - 5590 nt
p-value (RDP): 1,24E-14
MZ351172 - MW387995
MZ351172 - MK142237
MW387995 - MK142237
MZ351170
recombined fragment: 5579 - 5590 nt
p-value (RDP): 2,46E-02
MZ351172 - MW387995
MZ351172 - MZ351170
MW387995 - MZ351170
MZ351169
recombined fragment: 1298 – 1600 nt
p-value (RDP): 5,14E-03
MW387996 - MW387995
MW387996 - MZ351169
MW387995 - MZ351169
MZ351171
recombined fragment: 889 – 1600 nt
p-value (RDP): 1,15E-02
MW387996 - MW387995
MW387996 - MZ351171
MW387995 - MZ351171
Pairwise identity
Position in alignment

C)

Breakpoint 1298nt (P1)

non-recombined left part of P1

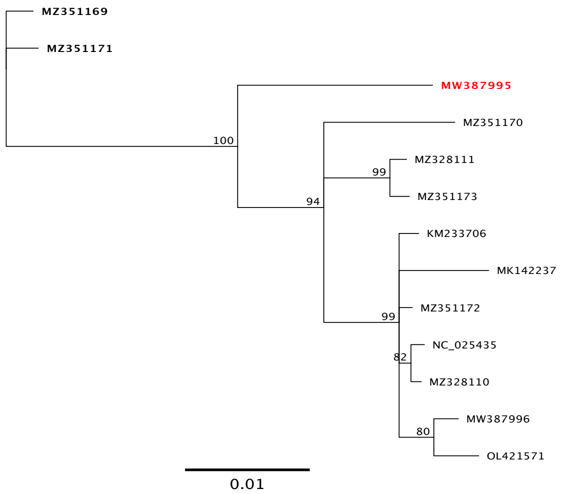

recombined middle part of P1

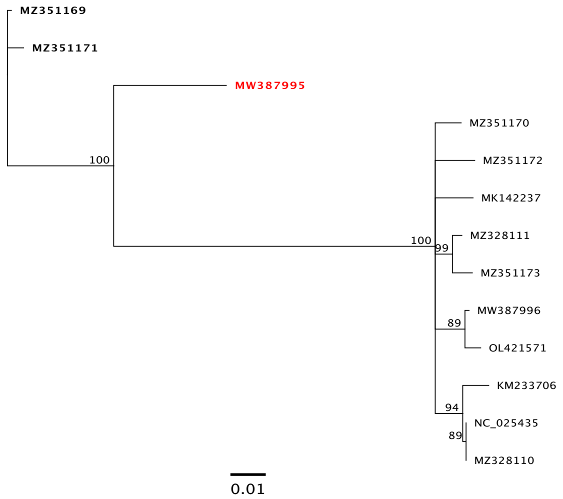

non-recombined right part of P1

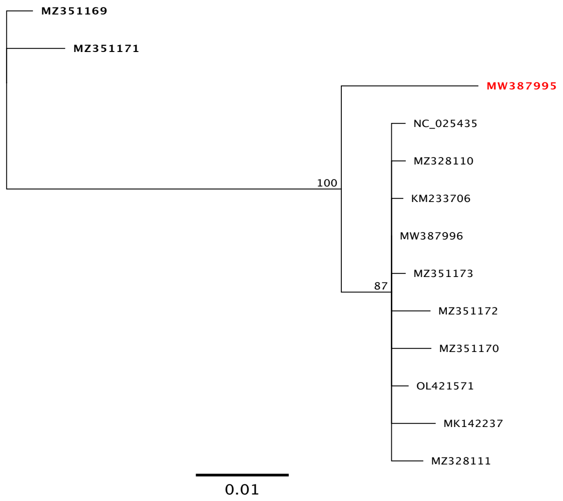

Breakpoint 5276nt (P5)

non-recombined left part of P5

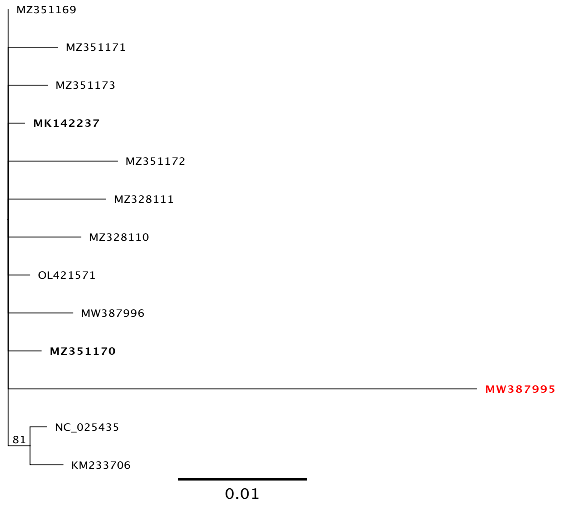

recombined right part of P5

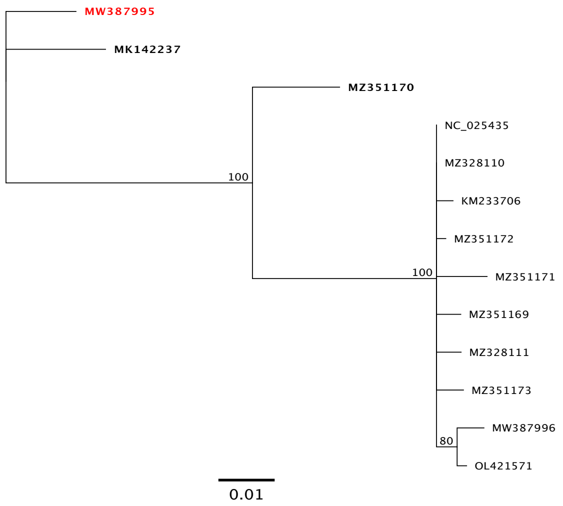

TableS1. Overview ofthe tested plants: symptoms,virusdetection andSangersequencingof SPV1isolatesoriginatingfromstrawberry plantsfromdifferent localitiesof the CzechRepublic

Sample					RT–PCRtesting							Sequence	RelatedSPV1			
Locality	Sample designation	Plant tested	Symptoms	RNA (ng/µl)	SMoV	SCV	SMYEV	SVBV	StrV1	SPV1	NADH	GenBank Acc.No.	Identity% (nt compared)	isolate	GenBank Acc.No.	origin
South Bohemian Region																
SB–1–F	91/2017	F. ananassa cv Rumba	chlorosis	175,7	–	–	–	–	–	–	+					
SB–1–F	93/2017	F. ananassa cv Rumba	chlorosis	1206,3	–	+	–	–	–	+	+					
SB–1–F	95/2017	F. ananassa cv Rumba	dwarf, reddening, chlorosis	374,5	+	–	–	–	–	+	+	not deposited	100,0(221)	AB4101	KM233706	Canada
SB–1–F	96/2017	F. ananassa cv Rumba	reddening, chlorosis	227,1	–	+	–	–	–	–	+					
SB–1–F	98/2017	F. ananassa cv Rumba	necroses	63,7	–	–	–	–	–	–	+					
SB–1–F	100/2017	F. ananassa cv Rumba	reddening	375,9	–	+	–	–	–	–	+					
SB–2–F	35/2017	F. ananassa cv Darselect	reddening, chlorosis	79,9	–	–	–	–	–	+	+	MW387980	99,5 (1674)	AB4101	KM233706	Canada
SB–2–F	37/2017	F. ananassa cv Darselect	malformation	47,8	–	–	–	–	–	–	+					
SB–2–F	38/2017	F. ananassa cv Darselect	dwarf	189,8	–	–	+	–	–	–	+					
SB–2–F	39/2017	F. ananassa cv Darselect	malformation, vein clearing	592,3	–	+	–	–	–	–	+					
SB–2–F	40/2017	F. ananassa cv Darselect	reddening	474,3	–	–	–	–	–	–	+					
SB–2–F	41/2017	F. ananassa cv Darselect	no symptoms	406,6	–	+	–	–	–	–	+					
SB–2–F	14/2020	F. ananassa cv Darselect	vein clearing	53,1	–	–	–	–	–	+	+					
SB–2–F	15/2020	F. ananassa cv Darselect	no symptoms	73,5	–	–	–	–	–	–	+					
SB–2–F	16/2020	F. ananassa cv Darselect	chlorosis	81,6	–	–	–	–	–	+	+					
SB–2–F	17/2020	F. ananassa cv Darselect	chlorosis	39,1	–	–	–	–	–	–	+					
SB–2–F	18/2020	F. ananassa cv Darselect	chlorosis	119,4	–	–	–	–	–	+	+					
SB–2–F	19/2020	F. ananassa cv Darselect	no symptoms	90,6	–	–	–	–	–	–	+					
SB–2–F	20/2020	F. ananassa cv Darselect	chlorosis	76,3	–	–	–	–	–	–	+					
SB–2–F	21/2020	F. ananassa cv Darselect	dwarf	147,8	–	–	–	–	–	+	+					
SB–2–F	22/2020	F. ananassa cv Darselect	dwarf	49,4	–	–	–	–	–	+	+					
SB–2–F	23/2020	F. ananassa cv Darselect	dwarf, malformation	38,3	–	–	–	–	–	+	+	MW387994	99.5 (1674)	AB4101	KM233706	Canada
SB–2–F	24/2020	F. ananassa cv Darselect	malformation, reddening	50,4	–	–	–	–	–	–	+					
SB–2–F	25/2020	F. ananassa cv Darselect	dwarf, malformation	140,4	–	–	–	–	–	+	+					
SB–2–F	28/2020	F. ananassa cv Darselect	chlorosis	82,3	–	–	–	–	–	+	+					
SB–2–F	31/2020	F. ananassa cv Darselect	no symptoms	104,3	–	–	–	–	–	–	+					
SB–2–F	32/ 2020	F. ananassa cv Darselect	dwarf, malformation	174,0	–	–	–	–	–	+	+					
SB–3–F	86/2017	F. ananassa cv Elkat	mosaic	741,0	–	+	–	–	+	–	+					
SB–3–F	88/2017	F. ananassa cv Elkat	dwarf	967,3	–	+	+	–	+	–	+					
SB–3–F	89/2017	F. ananassa cv Elkat	mosaic	95,5	–	–	–	–	+	–	+					
SB–3–F	90/2017	F. ananassa cv Elkat	dwarf, vein clearing	432,5	–	–	–	–	–	–	+					
SB–3–F	102/2017	F. ananassa cv Elkat	mosaic	50,2	–	–	–	–	+	–	+					
SB–3–F	103/2017	F. ananassa cv Elkat	dwarf	51,0	–	–	–	–	+	–	+					
SB–3–F	138/2017	F. ananassa cv Elkat	vein clearing		–	–	+	–	+	–	+					
SB–4–F	43/2017	F. ananassa cv Rumba	chlorosis	170,3	–	–	–	–	+	–	+					
SB–4–F	45/2017	F. ananassa cv Rumba	chlorosis	59,9	–	–	+	–	–	–	+					
SB–4–F	47/2017	F. ananassa cv Rumba	chlorosis	271,1	–	–	–	–	–	–	+					
SB–4–F	48/2017	F. ananassa cv Rumba	chlorosis	541,3	–	–	–	–	–	–	+					
SB–4–F	49/2017	F. ananassa cv Vesna	malformation	48,8	–	–	–	–	–	–	+					
SB–4–F	50/2017	F. ananassa cv Honeoye	malformation	85,9	–	–	–	–	–	–	+					
SB–4–F	51/2017	F. ananassa cv Honeoye	dwarf	92,3	–	–	+	–	–	–	+	not deposited	99,6(232)	AB4101	KM233706	Canada
SB–4–F	54/2017	F. ananassa cv Honeoye	chlorosis	73,8	–	–	+	–	–	–	+					
SB–5–F	55/2017	F. ananassa cv Flair	reddening	442,1	+	–	+	–	–	+	+					
SB–5–F	56/2017	F. ananassa cv Flair	malformation, chlorosis	831,4	+	–	+	–	–	–	+					
SB–5–F	57/2017	F. ananassa cv Flair	dwarf	76,8	–	+	+	–	–	–	+					
SB–5–F	58/2017	F. ananassa cv Symphony	reddening	255,3	–	–	–	–	–	–	+					
SB–5–F	59/2017	F. ananassa cv Senga Sengana	no symptoms	355,1	–	–	–	–	–	–	+					
SB–5–F	60/2017	F. ananassa cv Senga Sengana	no symptoms	544,4	–	–	–	–	–	–	+					
SB–5–F	61/2017	F. ananassa cv Kama	chlorosis	17,0	–	–	+	–	–	–	+					
SB–5–F	62/2017	F. ananassa cv unknown	dwarf	56,6	–	–	+	–	–	–	+					
SB–5–F	64/2017	F. ananassa cv Flair	reddening	149,1	–	–	–	–	–	–	+					
SB–5–F	66/2017	F. ananassa cv Flair	malformation, mosaic	999,3	–	–	–	–	–	+	+					
SB–6–F	67/2017	F. ananassa cv Sonata	dwarf	34,9	–	–	–	–	–	–	+					
SB–6–F	68/2017	F. ananassa cv Sonata	dwarf	52,2	–	–	–	–	–	–	+					
SB–6–F	69/2017	F. ananassa cv Sonata	reddening	66,3	–	–	–	–	–	–	+					
SB–6–F	70/2017	F. ananassa cv Karmen	reddening	60,7	–	+	–	–	–	–	+					
SB–6–F	71/2017	F. ananassa cv Florence	dwarf	568,1	–	–	–	–	–	–	+					
SB–6–F	72/2017	F. ananassa cv Sonata	dwarf	48,4	–	–	–	–	–	–	+					
SB–6–F	73/2017	F. ananassa cv Sonata	dwarf	28,1	–	+	–	–	–	+	+					
SB–6–F	74/2017	F. ananassa cv Sonata	dwarf	29,9	–	+	–	–	–	+	+					
SB–6–F	75/2017	F. ananassa cv Florence	dwarf	704,9	–	–	–	–	–	–	+					
SB–6–F	76/2017	F. ananassa cv Florence	dwarf	147,4	–	–	–	–	–	–	+					
SB–6–F	77/2017	F. ananassa cv Salsa	dwarf	111,4	–	–	+	–	–	–	+					
SB–7–G	2/2016	F. ananassa cv unknown	malformation	310,5	–	+	–	–	–	–	+					
SB–7–G	30/2016	F. ananassa cv unknown	dwarf	280,1	–	–	–	–	+	–	+					
SB–7–G	**34/2016**	F. ananassa cv unknown	dwarf, malformation	266,0	–	–	–	–	+	+	+	**MW387996**	99,4 (2354)	AB4101	KM233706	Canada
SB–7–G	35/2016(I)	F. ananassa cv unknown	dwarf, chlorosis	240,4	–	–	+	–	+	–	+					
SB–7–G	36/2016(II)	F. ananassa cv unknown	dwarf	198,6	–	–	–	–	–	–	+					
SB–7–G	45/2016	F. ananassa cv unknown	dwarf	278.3	–	–	–	–	+	+	+					
SB–7–G	185/2021	F. ananassa cv unknown	chlorosis	294,6	+	–	–	–	+	–	+					
SB–7–G	186/2021	F. ananassa cv unknown	dwarf, malformation, chlorosis	302,8	–	–	+	–	+	–	+					
SB–7–G	45/2019	F. ananassa cv unknown	dwarf, malformation,chlorosis	299,9	+	+	+	–	+	+	+					
SB–8–W	99/2020	F. vesca (wild)	necrosis	63,2	+	–	–	–	–	–	+					
SB–8–W	100/2020	F. vesca (wild)	necrosis	29,5	+	–	–	–	–	–	+					
SB–8–W	101/2020	F. vesca (wild)	dwarf, malformation, necrosis	82,4	+	–	–	–	–	–	+					
SB–9–G	47/2016	F. vesca (wild)	chlorosis	280,9	–	+	–	–	–	–	+					
SB–9–G	48/2016	F. vesca (wild)	chlorosis	296,3	–	+	–	–	–	–	+					
SB–9–G	21/2017	F. vesca (wild)	mosaic	222,1	+	+	–	–	+	–	+					
SB–9–G	196/2017	F. ananassa cv unknown	vein clearing	885,8	+	+	–	–	+	–	+					
SB–9–G	197/2017	F. ananassa cv unknown	chlorosis	1256,6	+	+	–	–	–	–	+					
SB–10–G	**1/2017**	F. vesca semperflorenscv Rujana	dwarf, malformation, mosaic	50,0	+	+	–	–	+	+	+	**MW387995**	96,9 (2477)	SPV1/H2470	MZ351170	USA
SB–10–G	2/2017	F. vesca semperflorenscv Rujana	dwarf, malformation, mosaic	65,8	+	+	–	–	+	+	+					
SB–10–G	3/2017	F. vesca semperflorenscv Rujana	dwarf, malformation, mosaic	42,9	+	+	–	–	+	+	+					
SB–10–G	4/2017	F. vesca semperflorenscv Rujana	dwarf, malformation, mosaic	57,1	+	+	–	–	–	+	+					
SB–10–G	5/2017	F. vesca semperflorenscv Rujana	dwarf, malformation, mosaic	41,4	–	+	–	–	–	+	+					
SB–10–G	6/2017	F. vesca semperflorenscv Rujana	dwarf, malformation, mosaic	74,1	+	+	+	–	+	+	+					
SB–10–G	7/2017	F. vesca semperflorenscv Rujana	dwarf, malformation, mosaic	119,6	+	+	+	–	+	+	+	MW387977	97,3 (1679)	SPV1/H2470	MZ351170	USA
SB–10–G	8/2017	F. vesca semperflorenscv Rujana	dwarf, malformation, mosaic	32,2	+	+	–	–	–	+	+					
SB–10–G	9/2017	F. vesca semperflorenscv Rujana	dwarf, malformation, mosaic	178,9	–	+	+	–	–	+	+					
SB–10–G	10/2017	F. vesca semperflorenscv Rujana	dwarf, malformation, mosaic	624,9	+	+	–	–	+	+	+					
SB–10–G	11/2017	F. vesca semperflorenscv Rujana	dwarf, malformation, mosaic	69,5	+	+	–	–	+	+	+					
SB–10–G	1/2019_t*	F. vesca semperflorenscv Rujana	dwarf, mosaic	134,2	–	–	–	–	+	–	+					
SB–10–G	2/2019_t*	F. vesca semperflorenscv Rujana	no symptoms	48,0	–	+	–	–	+	+	+					
SB–10–G	3/2019_t*	F. vesca semperflorenscv Rujana	no symptoms	26,9	–	–	–	–	–	–	+					
SB–10–G	4/2019_t*	F. vesca semperflorenscv Rujana	no symptoms	48,5	–	–	–	–	–	–	+					
SB–10–G	5/2019_t*	F. vesca semperflorenscv Rujana	no symptoms	36,9	+	–	–	–	–	–	+					
SB–11–G	13/2017	F. ananassa cv unknown	reddening	13,1	–	–	–	–	–	–	+					
SB–11–G	14/2017	F. ananassa cv unknown	malformation	7,1	–	+	–	–	–	–	+					

SB–11–G	15/2017	F. ananassa cv unknown	malformation	2,6	–	–	–	–	–	–	+					
SB–11–G	16/2017	F. ananassa cv unknown	malformation	2,0	–	–	–	–	–	–	+					
SB–11–G	17/2017	F. ananassa cv unknown	malformation	14,0	–	+	–	–	–	–	+					
SB–12–W	J1/2021	F. vesca (wild)	no symptoms	30,2	+	–	–	–	+	–	+					
SB–12–W	J2/2021	F. vesca (wild)	no symptoms	39,7	+	–	–	–	+	–	+					
SB–12–W	J3/2021	F. vesca (wild)	no symptoms	94,3	–	–	–	–	–	–	+					
SB–12–W	J4/2021	F. vesca (wild)	no symptoms	58,0	–	–	–	–	+	–	+					
SB–12–W	J5/2021	F. vesca (wild)	no symptoms	24,5	–	–	–	–	–	–	+					
SB–12–W	J6/2021	F. vesca (wild)	no symptoms	68,3	+	–	–	–	–	–	+					
SB–12–W	J7/2021	F. vesca (wild)	no symptoms	6,3	+	–	–	–	–	–	+					
SB–13–W	J8/2021	F. vesca (wild)	no symptoms	18,9	–	–	–	–	–	–	+					
SB–13–W	J9/2021	F. vesca (wild)	no symptoms	88,0	–	–	–	–	–	–	+					
SB–13–W	J10/2021	F. vesca (wild)	no symptoms	13,0	–	–	–	–	–	–	+					
SB–13–W	J11/2021	F. vesca (wild)	no symptoms	60,1	–	–	–	–	–	–	+					
SB–13–W	J12/2021	F. vesca (wild)	no symptoms	28,9	–	–	–	–	–	–	+					
SB–13–W	J13/2021	F. vesca (wild)	no symptoms	56,8	–	–	–	–	–	–	+					
SB–13–W	J14/2021	F. vesca (wild)	no symptoms	3,2	–	–	–	–	–	–	+					
Pilsen Region																
P–1–F	115/2017	F. ananassa cv Christine	malformation, chlorosis		–	–	–	–	–	–	+					
P–1–F	116/2017	F. ananassa cv Christine	chlorosis	255,6	–	–	–	–	–	+	+	MW387981	99,4 (1666)	AB4101	KM233706	Canada
P–1–F	117/2017	F. ananassa cv Christine	chlorosis	353,8	–	+	–	–	–	–	+					
P–1–F	118/2017	F. ananassa cv Christine	chlorosis	411,2	–	–	–	–	–	–	+					
P–1–F	120/2017	F. ananassa cv Asia	dwarf	460,9	+	+	–	–	–	–	+					
P–1–F	121/2017	F. ananassa cv Asia	malformation	297,2	+	+	–	–	–	+	+					
P–1–F	127/2017	F. ananassa cv Asia	dwarf, chlorosis	188,6	–	–	–	–	–	+	+					
P–1–F	128/2017	F. ananassa cv Christine	dwarf	189,4	+	+	–	–	–	+	+					
P–1–F	151/2020	F.ananassa cv Daroyal	chlorosis	134,4	+	+	+	–	–	+	+					
P–1–F	152/2020	F.ananassa cv Daroyal	dwarf	122,2	+	+	+	–	–	+	+					
P–1–F	153/2020	F.ananassa cv Daroyal	chlorosis	31,7	+	+	+	–	–	+	+					
P–1–F	154/2020	F.ananassa cv Daroyal	chlorosis	76,7	+	+	+	–	–	–	+					
P–1–F	155/2020	F.ananassa cv Allegro	malformation	79,7	–	+	+	–	–	–	+					
P–1–F	156/2020	F.ananassa cv Allegro	malformation	143,1	+	+	+	–	–	+	+					
P–1–F	157/2020	F.ananassa cv Allegro	no symptoms	149,0	–	+	+	–	–	–	+					
P–1–F	158/2020	F.ananassa cv Allegro	dwarf	53,4	–	+	+	–	–	+	+					
P–1–F	159/2020	F.ananassa cv Allegro	chlorosis	34,2	+	+	–	–	–	+	+					
P–1–F	160/2020	F.ananassa cv Allegro	dwarf, dying, reddening, chlorosis	90,1	–	+	+	–	–	+	+	OL421564	99,3 (1696)	AB4101	KM233706	Canada
P–1–F	161/2020	F.ananassa cv Allegro	dwarf, dying, reddening	87,7	+	+	+	–	–	+	+					
P–1–F	162/2020	F.ananassa cv Allegro	malformation, chlorosis	68,4	+	+	+	–	–	+	+					
P–1–F	163/2020*	F.ananassa cv Allegro	dwarf	145,2	–	+	+	–	–	–	+					
P–1–F	164/2020*	F.ananassa cv Allegro	dwarf	70,9	+	+	+	–	–	+	+					
P–1–F	165/2020*	F.ananassa cv Allegro	dwarf	48,2	+	+	+	–	–	+	+					
P–1–F	166/2020*	F.ananassa cv Laetitia	dwarf, malformation	53,5	+	+	+	–	–	+	+					
P–1–F	167/2020*	F.ananassa cv Laetitia	dwarf	24,1	+	+	+	–	–	+	+					
P–1–F	168/2020*	F.ananassa cv Laetitia	chlorosis	65,1	+	+	+	–	–	+	+					
P–1–F	169/2020*	F.ananassa cv Laetitia	malformation	130,8	+	+	+	–	–	+	+	OL421565	99,2 (1696)	AB4101	KM233706	Canada
P–1–F	170/2020*	F.ananassa cv Laetitia	malformation, chlorosis	36,9	+	+	+	–	–	+	+					
P–1–F	171/2020*	F. ananassa cv Asia	dwarf	74,6	+	+	+	–	–	+	+					
P–1–F	172/2020*	F. ananassa cv Asia	dwarf, reddening	63,6	+	+	+	–	–	+	+					
P–1–F	173/2020*	F. ananassa cv Asia	chlorosis	124,7	–	+	+	–	–	+	+					
P–1–F	174/2020*	F. ananassa cv Asia	chlorosis	78,0	+	+	+	–	–	+	+					
P–1–F	175/2020*	F. ananassa cv Asia	no symptoms	170,7	–	+	–	–	–	+	+					
P–1–F	176/2020*	F. ananassa cv Albion	dwarf, reddening	48,3	–	+	+	–	–	+	+					
P–1–F	177/2020*	F. ananassa cv Daroyal	dwarf	64,8	+	+	+	–	–	+	+					
P–1–F	178/2020*	F. ananassa cv Magnus	dwarf	100,3	–	+	+	–	–	+	+					
P–1–F	179/2020*	F. ananassa cv FE1711	chlorosis	66,1	–	+	+	–	–	+	+					
P–1–F	180/2020*	F. ananassa cv FE1711	dwarf, chlorosis	61,0	+	+	+	–	–	+	+					
P–1–F	181/2020*	F. ananassa cv FE1711	dwarf, reddening	34,4	+	+	+	–	–	+	+					
P–2–F	79/2017	F. ananassa cv Elkat	dwarf	937,7	–	+	+	–	–	–	+					
P–2–F	80/2017	F. ananassa cv Elkat	dwarf	281,5	–	–	–	–	–	–	+					
P–2–F	82/2017	F. ananassa cv Elkat	necrosis, vein clearing	885,6	–	–	+	–	–	–	+					
P–2–F	83/2017	F. ananassa cv Elkat	dwarf, vein clearing	367,7	–	–	–	–	+	–	+					
P–2–F	84/2017	F. ananassa cv Elkat	dwarf	353,8	–	+	+	–	–	–	+					
P–3–F	131/2017	F. ananassa cv Darselect	dwarf	144,7	–	–	–	–	–	+	+					
P–3–F	132/2017	F. ananassa cv Darselect	chlorosis	246,3	–	–	–	–	–	+	+	MW387982	99,1 (1627)	AB4101	KM233706	Canada
P–4–F	134/2017	F. ananassa cv Florence	dwarf	158,8	–	–	–	–	–	–	+					
P–4–F	136/2017	F. ananassa cv Darselect	vein clearing	128,1	–	–	+	–	–	+	+	MW387983	99,4 (1669)	AB4101	KM233706	Canada
Liberec Region																
L–1–N	19/2016	F. ananassa cv unknown	mosaic	157,8	+	–	–	–	–	+	+					
L–1–N	T22/2016	F. ananassa cv unknown	chlorosis	176,3	–	–	–	–	–	+	+	MW387978	99,2 (1693)	AB4101	KM233706	Canada
L–1–N	27/2016	F. ananassa cv Elkat	necroses, vein clearing	54,2	–	–	–	–	+	–	+					
Hradec Králové Region																
HK–1–F	26/2016	F. ananassa cv unknown	dwarf, chlorosis		–	–	–	–	–	+	+					
South Moravian Region																
SM–1–F	173/2017	F. ananassa cv Prima	dwarf	430,0	–	–	–	–	–	–	+					
SM–1–F	176/2017	F. ananassa cv Prima	dwarf	1070,2	–	–	–	–	–	+	+	not deposited	100,0(222)	AB4101	KM233706	Canada
SM–1–F	179/2017	F. ananassa cv Elkat	malformation	270,9	–	–	–	–	–	–	+					
SM–1–F	181/2017	F. ananassa cv Elkat	chlorosis	647,2	–	+	–	–	–	–	+					
SM–1–F	182/2017	F. ananassa cv Elkat	chlorosis	251,1	–	–	–	–	–	–	+					
SM–1–F	183/2017	F. ananassa cv Elkat	malformation, necroses,vein clearing	324,6	–	+	+	–	–	+	+					
SM–2–F	154/2017	F. ananassa cv unknown	dwarf, chlorosis	253,1	–	–	–	–	–	+	+					
SM–2–F	155/2017	F. ananassa cv unknown	dwarf, chlorosis	604,8	–	–	–	–	–	–	+					
SM–2–F	156/2017	F. ananassa cv unknown	malformation	374,8	–	–	–	–	–	–	+					
SM–2–F	157/2017	F. ananassa cv Karmen	no symptoms	426,0	–	–	–	–	–	–	+					
SM–2–F	158/2017	F. ananassa cv unknown	malformation	243,6	–	–	–	–	–	–	+					
SM–2–F	162/2017	F. ananassa cv unknown	dwarf	312,3	–	–	+	–	–	–	+					
SM–2–F	163/2017	F. ananassa cv unknown	chlorosis	273,5	–	–	–	–	–	–	+					
SM–3–F	150/2017	F. ananassa cv unknown	chlorosis	336,7	–	–	–	–	–	–	+					
SM–3–F	151/2017	F. ananassa cv unknown	dwarf, chlorosis	1806,2	–	–	–	–	–	–	+					
SM–3–F	152/2017	F. ananassa cv unknown	dwarf	186,4	–	–	–	–	+	–	+					
SM–3–F	153/2017	F. ananassa cv unknown	dwarf	127,0	–	+	–	–	–	–	+					
SM–4–F	184/2017	F. ananassa cv Darselect	chlorosis	240,9	–	–	–	–	–	–	+					
SM–4–F	185/2017	F. ananassa cv Darselect	dwarf	512,8	–	+	–	–	–	+	+	MW387984	99,4 (1601)	AB4101	KM233706	Canada
SM–4–F	187/2017	F. ananassa cv Sonata	dwarf	580,8	–	+	–	–	–	–	+					
SM–4–F	188/2017	F. ananassa cv Sonata	dwarf	111,1	–	–	–	–	–	–	+					
SM–4–F	189/2017	F. ananassa cv Darselect	dwarf	130,2	–	–	–	–	–	–	+					
SM–4–F	190/2017	F. ananassa cv Darselect	reddening	866	–	–	–	–	–	+	+	not deposited	99,6(223)	AB4101	KM233706	Canada
SM–4–F	191/2017	F. ananassa cv Sonata	dwarf	468,1	–	–	+	–	–	+	+					
SM–4–F	194/2017	F. ananassa cv Sonata	chlorosis	214,7	–	–	–	–	–	–	+					
SM–5–F	2/2019	F. ananassa cv Darselect	reddening	120,5	–	–	+	–	–	+	+					
SM–5–F	3/2019	F. ananassa cv Darselect	no symptoms	208,8	–	–	–	–	–	+	+					

SM-5-F	4/2019	F. ananassa cv Honeoye	dwarf	224	–	–	–	–	–	–	+					
SM-5-F	5/2019	F. ananassa cv Honeoye	no symptoms	128,7	–	–	–	–	–	–	+					
SM-5-F	6/2019	F. ananassa cv Honeoye	dwarf	181,8	–	–	–	–	–	–	+					
SM-5-F	7/2019	F. ananassa cv Honeoye	chlorosis	229,5	–	–	–	–	–	–	+					
SM-5-F	8/2019	F. ananassa cv Allegro	no symptoms	144,8	–	–	–	–	–	–	+					
SM-5-F	9/2019	F. ananassa cv Symphony	dwarf, dying, reddening	267,2	–	–	–	–	–	+	+	MW387990	99,1 (1696)	AB4101	KM233706	Canada
SM-5-F	10/2019	F. ananassa cv Symphony	dying, malformation, chlorosis	228,8	–	–	–	–	–	+	+					
SM-5-F	11/2019	F. ananassa cv Roxana	dying, reddening, chlorosis	105,8	–	–	–	–	–	–	+					
SM-5-F	12/2019	F. ananassa cv Roxana	no symptoms	172	–	–	–	–	–	–	+					
SM-5-F	13/2019	F. ananassa cv Darselect	dwarf, chlorosis	220,6	–	–	–	–	–	+	+					
SM-5-F	14/2019	F. ananassa cv Darselect	dying, reddening, chlorosis	163,5	–	–	–	–	–	+	+					
SM-5-F	15/2019	F. ananassa cv Christine	dwarf, malformation,necrosis, chlorosis	376	–	–	–	–	–	–	+					
SM-5-F	16/2019	F. ananassa cv Christine	mosaic, necrosis	218,3	–	–	–	–	–	–	+					
SM-5-F	99/2019	F. ananassa cv Symphony	dying, reddening, chlorosis	166,7	+	–	–	–	–	–	+					
SM-5-F	100/2019	F. ananassa cv Symphony	dying, reddening, chlorosis	119,6	–	–	–	–	–	+	+	MW387993	99,1 (1696)	AB4101	KM233706	Canada
SM-6-F	165/2017	F. ananassa cv Rumba	malformation	475	–	+	–	–	–	–	+					
SM-6-F	166/2017	F. ananassa cv Rumba	dwarf	1535,5	–	–	+	–	–	+	+	not deposited	100,0(222)	AB4101	KM233706	Canada
SM-6-F	167/2017	F. ananassa cv Rumba	chlorosis	227,6	–	–	–	–	–	–	+					
SM-6-F	168/2017	F. ananassa cv Rumba	dwarf	399	–	–	–	–	–	–	+					
SM-6-F	170/2017	F. ananassa cv Rumba	dwarf	614,9	–	–	–	–	–	–	+					
SM-6-F	171/2017	F. ananassa cv Wendy	no symptoms	167,5	–	+	–	–	–	+	+					
SM-6-F	172/2017	F. ananassa cv Rumba	chlorosis	176,4	–	+	–	–	–	–	+					
SM-7-F	140/2017	F. ananassa cv Asia	chlorosis	316,7	–	–	–	–	–	–	+					
SM-7-F	143/2017	F. ananassa cv unknown	dwarf	419,3	–	–	–	–	–	–	+					
SM-7-F	144/2017	F. ananassa cv unknown	malformation	163,2	–	+	+	–	–	+	+	not deposited	100,0(222)	AB4101	KM233706	Canada
SM-7-F	145/2017	F. ananassa cv unknown	chlorosis	346,2	–	–	–	–	–	–	+					
SM-7-F	147/2017	F. ananassa	chlorosis	456,7	–	–	+	–	+	–	+					
Olomouc Region																
O-1-F	102/2020	F. ananassa cv Rumba	dwarf, reddening	105,6	–	–	+	–	–	–	+					
O-1-F	103/2020	F. ananassa cv Rumba	no symptoms	46,8	–	–	–	–	–	–	+					
O-1-F	104/2020	F.ananassa cv Rumba	no symptoms	86,2	–	–	–	–	–	–	+					
O-1-F	105/2020	F.ananassa cv Rumba	dwarf	125,2	–	–	–	–	–	–	+					
O-1-F	106/2020	F.ananassa cv Rumba	chlorosis	163,9	–	–	–	–	–	–	+					
O-1-F	107/2020	F.ananassa cv Rumba	mosaic	140,2	–	+	–	–	–	–	+					
O-1-F	108/2020	F.ananassa cv Rumba	dwarf, reddening	129,5	–	–	–	–	–	–	+					
O-1-F	109/2020	F.ananassa cv Rumba	dwarf, mosaic	220,8	–	–	+	–	–	–	+					
O-1-F	110/2020	F.ananassa cv Rumba	dwarf	76,9	–	–	–	–	–	–	+					
O-1-F	111/2020	F.ananassa cv Rumba	dwarf	100,7	–	–	–	–	–	–	+					
O-1-F	112/2020	F.ananassa cv Salsa	no symptoms	117,2	–	–	–	–	–	–	+					
O-1-F	113/2020	F.ananassa cv Rumba	dwarf, mosaic, reddening	128,7	–	–	–	–	–	–	+					
O-1-F	114/2020	F.ananassa cv Salsa	dwarf, malformation, chlorosis	95,9	–	–	–	–	–	–	+					
O-1-F	115/2020	F.ananassa cv Honeoye	dwarf	176	–	–	–	–	–	–	+					
O-1-F	116/2020	F.ananassa cv Honeoye	dwarf	105,7	+	–	–	–	–	–	+					
O-1-F	117/2020	F.ananassa cv Salsa	no symptoms	121,1	–	–	–	–	–	–	+					
O-1-F	118/2020	F.ananassa cv Salsa	no symptoms	149,5	–	–	–	–	–	–	+					
O-1-F	119/2020	F.ananassa cv Salsa	no symptoms	191	–	–	–	–	–	–	+					
O-1-F	120/2020	F.ananassa cv Salsa	no symptoms	329,5	–	–	–	–	–	–	+					
O-1-F	121/2020	F.ananassa cv Daroyal	no symptoms	106,3	–	–	–	–	–	–	+					
O-1-F	122/2020	F.ananassa cv Daroyal	mosaic	159,6	–	–	–	–	–	–	+					
O-1-F	123/2020	F.ananassa cv Daroyal	malformation, chlorosis	110,4	–	–	–	–	–	–	+					
O-1-F	124/2020	F.ananassa cv Sonata	dwarf, dying, reddening, chlorosis	70,5	–	–	–	–	–	–	+					
O-1-F	125/2020	F.ananassa cv Sonata	dying, reddening, chlorosis	60	–	–	–	–	–	–	+					
O-1-F	126/2020	F.ananassa cv Sonata	dying, reddening, chlorosis	33,5	–	–	–	–	–	–	+					
O-1-F	127/2020	F.ananassa cv Faith	no symptoms	118,6	–	–	–	–	–	–	+					
O-1-F	128/2020	F.ananassa cv Rumba	dwarf, malformation, reddening	101,2	–	–	–	–	–	–	+					
O-2-F	20/2019	F. ananassa cv Rumba	no symptoms	170,5	–	–	–	–	–	–	+					
O-2-F	22/2019	F. ananassa cv Rumba	vein clearing	249,8	–	–	–	–	+	–	+					
O-2-F	24/2019	F. ananassa cv Karmen	vein clearing	115,9	+	–	–	–	+	–	+					
O-2-F	26/2019	F. ananassa cv Rumba	dwarf, malformation	113,9	–	–	–	–	–	–	+					
O-2-F	27/2019	F. ananassa cv Rumba	vein clearing	107,3	–	–	–	–	+	–	+					
O-2-F	28/2019	F. ananassa cv Honeoye	vein clearing	124	–	–	–	–	+	–	+					
Zlín Region																
Z-1-F	31/2019	F. ananassa cv Rumba	no symptoms	175,1	–	–	–	–	–	–	+					
Z-1-F	32/2019	F. ananassa cv Rumba	dying, malformation, reddening	87,7	–	–	–	–	–	–	+					
Z-1-F	33/2019	F. ananassa cv Rumba	dying, malformation, reddening	126	–	–	–	–	–	–	+					
Z-1-F	34/2019	F. ananassa cv Rumba	dwarf	204,1	–	–	–	–	–	–	+					
Z-1-F	35/2019	F. ananassa cv Alegro	no symptoms	93,6	–	–	–	–	–	–	+					
Z-1-F	36/2019	F. ananassa cv Allegro	dying, malformation, reddening	163,6	–	–	–	–	–	–	+					
Z-1-F	37/2019	F. ananassa cv Allegro	no symptoms	306,2	–	–	–	–	–	–	+					
Z-1-F	38/2019	F. ananassa cv Asia	dying, malformation, reddening	151,2	–	–	–	–	–	–	+					
Z-1-F	39/2019	F. ananassa cv Asia	dying, malformation, chlorosis	195,9	–	–	–	–	–	–	+					
Z-1-F	40/2019	F. ananassa cv Asia	dying, malformation, chlorosis	203,6	–	–	–	–	–	–	+					
Z-1-F	41/2019	F. ananassa cv Salsa	dwarf	176,3	–	–	–	–	–	–	+					
Z-1-F	43/2019	F. ananassa cv Salsa	dying, malformation, reddening	269,1	–	–	–	–	–	–	+					
Z-1-F	44/2019	F. ananassa cv Salsa	dwarf	173,8	–	–	–	–	–	–	+					
Z-1-F	50/2019	F. ananassa cv Claire	dying, malformation, chlorosis	99,6	–	+	–	–	–	–	+					
Z-1-F	101/2019	F. ananassa cv Rumba	no symptoms	293,8	+	–	–	–	–	–	+					
Z-1-F	102/2019	F. ananassa cv Salsa	no symptoms	293,6	–	–	–	–	–	–	+					
Z-1-F	103/2019	F. ananassa cv Salsa	dying, malformation, reddening	127,7	–	–	–	–	–	–	+					
Z-1-F	104/2019	F. ananassa cv Renesance	no symptoms	139,8	–	–	–	–	–	–	+					
Z-1-F	105/2019	F. ananassa cv Renesance	dwarf,dying, malformation, reddening	155,9	–	–	–	–	–	–	+					
Z-1-F	106/2019	F. ananassa cv Rumba	dying, malformation, reddening	141,5	+	–	–	–	–	–	+					
Z-1-F	130/2020	F. ananassa cv Destina	dying, malformation, reddening	296,4	–	–	–	–	–	–	+					
Z-1-F	131/2020	F. ananassa cv Rumba	dying, malformation, reddening	278,8	–	–	+	–	–	–	+					
Z-2-F	52/2019	F. ananassa cv. Lesana	dwarf	164	–	–	–	–	–	–	+					
Z-2-F	53/2019	F. ananassa cv. Lesana	no symptoms	110,5	–	–	–	–	–	–	+					
Z-2-F	54/2019	F. ananassa cv. Lesana	no symptoms	228,9	–	–	–	–	–	–	+					
Z-2-F	55/2019	F. ananassa cv. Lesana	dwarf	360,8	–	–	–	–	–	–	+					
Z-2-F	56/2019	F. ananassa cv. Lesana	no symptoms	67,9	–	–	–	–	–	–	+					
Moravian–Silesian Region																
MS-1-F	84/2019	F. ananassa cv Elkat	vein clearing	229	+	–	+	–	+	–	+					
MS-1-F	85/2019	F. ananassa cv Elkat	mosaic, vein clearing	144,1	+	–	–	–	+	+	+	MW387992	99,3 (1692)	AB4101	KM233706	Canada
MS-1-F	86/2019	F. ananassa cv Elkat	vein clearing	223,3	+	–	–	–	–	+	+					
MS-1-F	88/2019	F. ananassa cv Elkat	mosaic	196,6	+	–	–	–	–	–	+					
MS-1-F	89/2019	F. ananassa cv Elkat	no symptoms	236,6	+	–	–	–	+	+	+					
MS-1-F	90/2019	F. ananassa cv Elkat	vein clearing	154,9	+	–	–	–	+	+	+					
MS-1-F	91/2019	F. ananassa cv Elkat	malformation, vein clearing	181,5	+	–	–	–	+	–	+					
MS-1-F	92/2019	F. ananassa cv Elkat	mosaic,vein clearing	168,8	+	–	+	–	+	–	+					
MS-1-F	93/2019	F. ananassa cv Elkat	mosaic, vein clearing	98,9	+	–	–	–	+	–	+					
MS-1-F	94/2019	F. ananassa cv Elkat	dwarf, vein clearing	123,4	+	–	–	–	+	+	+					
MS-1-F	96/2019	F. ananassa cv Elkat	malformation, vein clearing	149,1	+	–	–	–	+	–	+					
MS-1-F	97/2019	F. ananassa cv Elkat	mosaic	259,4	+	–	–	–	+	–	+					
MS-1-F	98/2019	F. ananassa cv Elkat	dwarf, mosaic	182,4	+	–	+	–	+	–	+					

MS–2–F	57/2019	F. ananassa cv Elkat	vein clearing	243,5	+	–	+	–	+	+	+					
MS–2–F	58/2019	F. ananassa cv Elkat	vein clearing	167,9	+	–	+	–	+	+	+					
MS–2–F	59/2019	F. ananassa cv Elkat	no symptoms	148,7	+	+	+	–	+	+	+					
MS–2–F	60/2019	F. ananassa cv Elkat	dwarf	105,3	+	+	+	–	+	+	+					
MS–2–F	61/2019	F. ananassa cv Darselect	dwarf, chlorosis	109,9	+	+	+	–	–	+	+					
MS–2–F	62/2019	F. ananassa cv Faith	malformation	194,9	+	+	+	–	–	+	+					
MS–2–F	63/2019	F. ananassa cv Darselect	malformation, chlorosis	129,2	+	+	+	–	–	+	+					
MS–2–F	64/2019	F. ananassa cv Darselect	chlorosis	159,4	+	+	+	–	–	+	+					
MS–2–F	65/2019	F. ananassa cv Faith	chlorosis	138,4	+	+	+	–	–	+	+					
MS–2–F	66/2019	F. ananassa cv Faith	malformation	236,8	+	+	+	–	–	+	+	MW387991	99,3 (1644)	AB4101	KM233706	Canada
MS–2–F	67/2019	F. ananassa cv Faith	malformation, chlorosis	142,5	+	–	+	+	–	+	+	MW387997	SVBV	n.a.	n.a.	n.a.
MS–2–F	68/2019	F. ananassa cv Elkat	no symptoms	132,4	+	–	+	–	+	+	+					
MS–2–F	70/2019	F. ananassa cv Elkat	mosaic	167,9	+	–	+	–	+	+	+					
MS–2–F	71/2019	F. ananassa cv Elkat	dwarf, mosaic, necrosis	116,5	+	–	+	–	+	+	+					
MS–2–F	72/2019	F. ananassa cv Elkat	reddening	128,4	+	–	+	–	+	+	+					
MS–2–F	132/2020*	F. ananassa cv Elkat	no symptoms	59,3	+	–	+	–	+	+	+					
MS–2–F	133/2020*	F. ananassa cv Elkat	no symptoms	119,3	+	–	+	–	+	+	+					
MS–2–F	134/2020*	F. ananassa cv Elkat	no symptoms	129,5	+	–	+	–	+	+	+					
MS–2–F	135/2020*	F. ananassa cv Elkat	no symptoms	128,6	+	–	+	–	+	+	+					
MS–2–F	136/2020*	F. ananassa cv Elkat	no symptoms	219,6	+	–	+	–	+	–	+					
MS–2–F	137/2020*	F. ananassa cv Elkat	mosaic	108,5	+	–	+	–	+	+	+					
MS–2–F	**138/2020***	F. ananassa cv Elkat	dwarf	250,6	+	–	+	–	+	+	+	**OL421571**	99,1 (5985)	19SP105	MZ328110	Canada
MS–2–F	139/2020*	F. ananassa cv Elkat	no symptoms	360,1	+	–	+	–	+	–	+					
MS–2–F	140/2020*	F. ananassa cv Elkat	mosaic	214,1	+	–	+	–	+	+	+					
MS–2–F	141/2020*	F. ananassa cv Elkat	dwarf, chlorosis	104,1	+	–	+	–	+	+	+					
MS–2–F	142/2020*	F. ananassa cv Elkat	dwarf, malformation, chlorosis	72,7	+	–	+	–	+	+	+					
MS–2–F	143/2020*	F. ananassa cv Elkat	malformation	82,0	+	–	+	–	+	+	+					
MS–2–F	144/2020*	F. ananassa cv Elkat	malformation	191,7	+	–	+	–	+	–	+					
MS–2–F	145/2020	F. ananassa cv Elkat	dwarf, mosaic	112,0	+	–	+	–	+	–	+					
MS–2–F	146/2020	F. ananassa cv Faith	dwarf	219,1	+	–	+	–	–	+	+					
MS–3–F	73/2019	F. ananassa cv Florence	no symptoms	156,8	+	–	–	–	–	–	+					
MS–3–F	75/2019	F. ananassa cv Florence	malformation	110,6	–	–	–	–	–	–	+					
MS–3–F	76/2019	F. ananassa cv Florence	no symptoms	115,4	–	–	–	–	–	–	+					
MS–3–F	77/2019	F. ananassa cv Florence	malformation	92,5	+	–	–	–	+	–	+					
MS–3–F	78/2019	F. ananassa cv Florence	chlorosis	93,5	–	–	–	–	–	–	+					
MS–3–F	79/2019	F. ananassa cv Florence	chlorosis	94,1	–	–	+	–	–	–	+					
MS–3–F	80/2019	F. ananassa cv Florence	no symptoms	58,5	–	–	–	–	–	–	+					
MS–3–F	81/2019	F. ananassa cv Florence	dying, malformation	79,5	–	–	+	–	–	–	+					

in bold: samplesselected for HTS
+=positive reaction in RT–PCR, –=no amplification observed
* new seedlings
Locality: SB =South Bohemia Region, P =Pilsen Region, L =Liberec Region, HK=Hradec Králové Region, SM =South Moravia Region, O =Olomouc Region, Z=Zlín Region, MS =Moravia–Silesian Region, F =field, W =wood, N =nursery, G =garden

Number of plant samples	332
–without symptoms	63
–symptomatic	269

Table S2. Description of all primers used in the study

Primer name	Target	Sequence, 5' –3', or reference	Amplicon size, bp	qPCR efficiency, R^2	Running conditions
3220	16S ribosomal RNA (16S)	AGAAACCAACCTGGCTTACA	119	92.3%<br>0.997	95 °Cfor 12 min, 45 cycles of [95 °Cfor 10 s, 60 °Cfor 20 s, 72 °Cfor 20s, plate reading] and dissociation curve analysis.
3219		GCGACCTCGATGTTGAATTA			
3212	Succinate dehydrogenase B (Sdhb)	CAAACCCTTTGGGCATGTTTT	110	94.9%<br>0.992	
3211		ACTCCAGAGATAAAGCTACAGC			
SPV1.2fw	SPV1	CCAACTAGCCAAGATCCCATCTGAAG	186	105%<br>0.993	
SPV1.2rv		AGGCCTACACCAGCATCTAACTCA			
SmoV_UTR–F02	SMoV, 3' UTR	AGCGACCACGACTGTGACAAAG	209	NA	94°Cfor 2 min<br>40 cycles of [94°Cfor 20 sec, 60°Cfor 20 sec and 68°Cfor 20 sec] and the final extension at 68°Cfor 5 min
SmoV_UTR–R01		TTGGRTCGTCACCTGAYCTCG			
SMYEV F02	SMYEV, 3' UTR	TAACCAACMCACTCTAGACCAGG	201	NA	95°Cfor 5min<br>45 cycles of 95°Cfor 20 sec, 58°Cfor 20 sec and 72°Cfor 20 sec, and the final extension at 72°Cfor 5 min
SMYEV R02		CCGAGTAGGGGTTGATGATTTC			
Polero40Rv	SPV1, P1/P2 fusion region	Luciani et al. 2016	1030	NA	94°Cfor 2 min<br>40 cycles of 94°Cfor 30 sec, 55°Cfor 30 sec and 72°Cfor 1 min, and the final extension at 72°Cfor 10 min"
Polero2Fw					
Polero47Fw	SPV1, P1/P2 fusion region	Luciani et al. 2016	897	NA	94°Cfor 5min<br>35 cycles of 94°Cfor 30 sec, 50°Cfor 1 min and 72°Cfor 2 min, and the final extension at 72°Cfor 10 min
Polero2Rv					
Polero 2Fw	SPV1, P1/P2 fusion region	Luciani et al. 2016	1808	NA	94°Cfor 2 min<br>40 cycles of 94°Cfor 30 sec, 55°Cfor 30 sec and 72°Cfor 1 min, and the final extension at 72°Cfor 10 min"
SPV–1.41R		CGGTAGTGCTTGTAGGCCTT			
SPV–1F	SPV1, P1/P2 fusion region	Thekke-Veetil and Tzanetakis, 2016		NA	94°C for 1 min<br>35 cycles of [94°Cfor 15 sec, 55°Cfor 35 sec, and 72°Cfor 35 sec]<br>and the final extension at 72°Cfor 3 min
SPV–1R					
Atropa Nad2.1a	mRNA, NADH dehydrogenase nad5	Thompson et al. 2003		NA	94°C for 1 min<br>35 cycles of [94°Cfor 15 sec, 55°Cfor 35 sec, and 72°Cfor 35 sec]<br>and the final extension at 72°Cfor 3 min
Atropa Nad2b					

NA –not applicable

References:

–Luciani, C. E., Celli, M. G., Merino, M. C., Perotto, M. C., Pozzi, E., Conci, V. C. (2016). First report of Strawberry polerovirus 1in Argentina. Plant Disease, 100(7), 1510.

–Thekke-Veetil, T., & Tzanetakis, I. E. (2016). First report of strawberry polerovirus-1in strawberry in the United States. Plant Disease, 100(4), 867–867.

–Xiang, Y., Bernardy, M., Bhagwat, B., Wiersma, P. A., DeYoung, R., & Bouthillier, M. (2015). The complete genomesequence of a new polerovirusin strawberry plantsfromeastern Canada showingstrawberry decline symptoms. Archivesof virology, 160(2), 553–556.

–Thompson, J.R.; Wetzel, S.; Klerks, M.M.; Vašková, D.; Schoen, C.D.; Špak, J.; Jelkmann, W. MultiplexRT–PCR detection of four aphid–borne strawberry virusesin Fragaria spp. in combination with a plant mRNA specific internal control. J. Virol. Methods2003, 111, 85–93.

Table S3. Results of RT-PCRdetermination of SPV1 in Aphisgossypiibatches and recipient Fragaria vesca'Alpine' plants after transmissiontrials.

Acquisition/ inoculation access period	Aphis gossypii batches				Fragaria vesca ´Alpine´ plants			
	1	2	3	4	1	2	3	4
10 min/10 min	–	–	–	–	–	–	–	–
10 min/4 h	–	–	–	–	–	–	–	–
10 min/24 h	–	–	–	–	–	–	–	–
10 min/48 h	–	–	–	–	–	–	–	–
4 h/10 min	+	–	–	–	–	–	–	–
4 h/4 h	–	–	–	–	–	–	–	–
4 h/24 h	+	–	–	–	–	–	–	–
4 h/48 h	–	–	–	–	–	–	–	–
24 h/10 min	+	–	–	–	–	–	–	–
24 h/4 h	+	–	–	–	–	–	–	–
24 h/24 h	+	+	–	–	–	–	–	–
24 h/48 h	–	–	–	–	–	–	–	–
48 h/10 min	+	+	+	+	–	–	–	–
48 h/4 h	+	+	+	–	–	–	–	–
48 h/24 h	+	+	+	+	–	–	–	–
48 h/48 h	+	+	–	–	–	–	–	–

positive (+) or negative (-) samples tested by RT-PCR assays using primer pair SPV-1F/SPV-1R

Table S4. Synonymous vs. nonsynonymous nucleotide mutations in putative recombinant regions of the P1 (A) and P5 (B) genes. Sequences were analysed using recombination points obtained by RDP5 software (889 – 1660 nt for P1, 5276 – 5990 nt for P5), nucleotide changes were evaluated against sequence NC_025435. Each column counts number of mutated codons in the 1st, 2nd, 3rd, or more nucleotide positions in the codon, respectively. Synonymous mutations on 3rd position sequences evaluated as recombinant are marked yellow. Last column shows the ratio of mutated codons with the exact same nucleotide composition as equivalent codons in Rujana isolate MW387995 (potential donor of the recombined sequence); the ratio was evaluated only for potentially recombinant sequences.

(A)	synonymous				nonsynonymous				the same mutated codons as in MW387995
	1st	*2nd*	*3rd*	*more*	*1st*	*2nd*	*3rd*	*more*	
OL421571	–	–	15	–	–	–	–	–	n.a.
KM233706	–	–	4	–	–	–	–	–	n.a.
MK142237	–	–	13	–	2	–	–	–	n.a.
MW387995	–	–	58	1	–	–	2	3	**64/64**
MW387996	–	–	11	–	1	–	–	–	n.a.
MZ328110	–	–	1	–	–	–	–	–	n.a.
MZ328111	–	–	21	–	–	–	–	–	n.a.
MZ351169	2	–	61	1	1	–	1	4	**40/70**
MZ351170	–	–	26	–	1	–	1	–	n.a.
MZ351171	2	–	61	1	–	–	1	5	**42/70**
MZ351172	–	–	13	–	–	–	–	–	n.a.
MZ351173	–	–	22	–	1	–	–	–	n.a.

(B)	synonymous				nonsynonymous				the same mutated codons as in MW387995
	1st	*2nd*	*3rd*	*more*	*1st*	*2nd*	*3rd*	*more*	
OL421571	–	–	2	–	–	–	–	–	n.a.
KM233706	–	–	2	–	–	2	–	–	n.a.
MK142237	1	–	41	1	3	1	2	1	**37/50**
MW387995	1	–	39	1	1	–	2	4	**48/48**
MW387996	–	–	5	–	–	–	–	–	n.a.
MZ328110	–	–	1	–	–	–	–	–	n.a.
MZ328111	–	–	3	–	–	–	–	–	n.a.
MZ351169	1	–	3	–	–	–	–	–	n.a.
MZ351170	1	–	24	–	3	–	–	1	**18/29**
MZ351171	–	–	4	–	–	–	–	1	n.a.
MZ351172	–	–	–	–	1	–	–	–	n.a.
MZ351173	–	–	2	–	–	–	–	1	n.a.
